# The efficacy and safety of GLP-1 receptor agonists in youth with type 2 diabetes: a meta-analysis

**DOI:** 10.1186/s13098-024-01337-5

**Published:** 2024-04-24

**Authors:** Louise Buonalumi Tacito Yugar, Luis Gustavo Sedenho-Prado, Isadora Maria Castilho da Silva Ferreira, Cleide Aparecida Moreira Silva, Andrei C. Sposito, Cintia Cercato

**Affiliations:** 1 School of Medical Sciences, State University of Campinas, Campinas, SP 13083-970 Brazil; 2 Atherosclerosis and Vascular Biology Laboratory (Atherolab), State University of Campinas, Campinas, Brazil; 3 Obesity Unit, Division of Endocrinology and Metabolism, University of São Paulo Medical School Hospital, São Paulo, Brazil

## Abstract

**Background:**

Glucagon-like peptide 1 receptor agonists have been proven to be effective in adults with diabetes and children with obesity. However, children with type 2 diabetes constitute an underrepresented subpopulation with limited treatment options. This meta-analysis aimed to determine more precise estimates of the efficacy and safety of glucagon-like peptide-1 agonists in pediatric type 2 diabetes mellitus.

**Methods:**

Three databases were searched (PubMed, Embase, and Cochrane Central Register of Controlled Trials) for trials published until the end of March 2024. The search indexing terms included 3 categories: [1] type 2 diabetes mellitus [2], youth, and [3] glucagon-like peptide-1 receptor agonist (GLP-1 RA). Randomized controlled trials in youth with type 2 diabetes (age ≤ 18 years) that assessed anthropometric and metabolic parameters were included. A total of 1119 nonduplicate studies were retrieved, and 137 full-text articles were screened. The data were analyzed using mean differences (MDs) with 95% CIs and odds ratios (ORs) with 95% CIs. For outcomes with low heterogeneity, a fixed-effects model was used. Otherwise, we applied a random effects model. Our outcomes were Hb1Ac, fasting blood glucose (FBG), blood pressure, weight, and side effects.

**Results:**

Five studies comprehending 415 children and adolescents were included. On average, GLP-1 RA reduced HbA1c levels (-1.01%; 95% CI, -1.26 to -0.76), fasting blood glucose levels (-1.88 mmol/L; 95% CI, -2.51 to -1.26), and body weight (-1.6 kg; 95% CI, -2.83 to -0.36). No significant reductions in systolic blood pressure (MD -0.19 mmHg; 95% CI, -3.9 to 3.52 mmHg) or diastolic blood pressure (MD 0.3 mmHg; 95% CI, -2.33 to 2.93 mmHg) were observed. Despite a higher incidence of side effects, withdrawal rates from the studies remained low.

**Conclusions:**

Within this specific population, GLP-1 RAs exhibit a notable association with substantial reductions in HbA1c, FBG, and body weight. The administration of these medications is concurrent with an elevated incidence of side effects, which are predominantly gastrointestinal and tolerable.

**Trial registration:**

PROSPERO identifier: CRD42023393020.

**Supplementary Information:**

The online version contains supplementary material available at 10.1186/s13098-024-01337-5.

## Introduction

In 2021, an estimated 41,600 new cases of youth-onset type 2 diabetes emerged worldwide [1]. By 2017, among 1,848,899 youths aged 10–19 years in the United States, 1,230 children were diagnosed with type 2 diabetes mellitus (T2DM) [2]. The pathophysiology of T2DM constitutes interactions among genetic, environmental, and metabolic factors well described in previous literature [3]. 

Recent studies indicate that the prevalence of end-organ damage is greater in young people diagnosed with T2DM than in those diagnosed with type 1 diabetes [4]. Therefore, early intervention to prevent disease progression is essential. Until recently, only metformin and insulin were used for treatment in the pediatric population. However, the latest research has demonstrated the efficacy of liraglutide, exenatide, and dulaglutide, which have been approved by the United States Food and Drug Administration for treatment in this population [3, 5, 6]. 

Glucagon-like peptide 1 (GLP-1) reduces food intake and leads to weight loss by increasing insulin secretion and delaying gastric emptying [7, 8]. . According to the American Diabetes Association’s Standards of Care 2024, if glycemic targets are no longer met with metformin (with or without long-acting insulin), GLP-1 receptor agonist (GLP-1 RA) therapy should be considered [9]. However, the efficacy and safety of GLP-1 RA in pediatric populations are still being studied in randomized, double-blind clinical trials. Therefore, this meta-analysis aimed to evaluate the efficacy and adverse events of GLP-1 RA used for treating T2DM in children and adolescents.

## Methods

### Protocol registration

This systematic review was registered in PROSPERO under registration no. CRD42023393020 on February 3rd, 2023.

### Eligibility criteria

Inclusion in this meta-analysis was restricted to studies that met all the following eligibility criteria: 1) randomized double-blind clinical trials or post hoc analyses of randomized double-blind clinical trials; 2) compared GLP-1 receptor agonists to placebo; 3) patients had type 2 diabetes mellitus; and 4) were aged between 10 years old and 18 years. In addition, studies were included only if they reported any of the clinical outcomes of interest. We excluded studies with 1) no control group, 2) adult patients (18 years old or older), 3) patients without type 2 diabetes, or 4) patients with type 1 diabetes mellitus.

### Search strategy and information sources

We systematically searched PubMed, Embase, and the Cochrane Central Register of Controlled Trials for trials published until the end of March 2024. The references from all included studies, previous systematic reviews and meta-analyses were also searched manually for any additional studies. Two authors independently extracted the data following predefined search criteria and quality assessment. Disagreements between authors were resolved by a third author.

### Study selection and data collection process

The results obtained from the search across the databases were imported into reference management software. After eliminating duplicate entries, records were subjected to a preliminary screening based on their titles and abstracts. Potentially eligible records underwent a full-text analysis with reasons for exclusion documented. Study selection was carried out independently by two reviewers, and any disparities were resolved through consultation with a third reviewer.

For data collection, two independent authors extracted study characteristics, participants’ demographics and baseline characteristics, and outcome data. Some reported data were not available in the published papers or supplementary appendices. In these cases, we manually searched the ClinicalTrials.gov register or the European Union Clinical Trials Register of the study. If they could still not be found, we requested them directly from the pharmaceutical sponsors. Efficacy outcomes included changes in glycated hemoglobin (HbA1c), fasting blood glucose (FBG), body weight, systolic blood pressure (SBP), and diastolic blood pressure (DBP). Outcomes regarding side effects included nausea, diarrhea, vomiting, abdominal pain, and hypoglycemia (any event of plasma glucose ≤ 3.9 mmol/L). We collected data from pooled analyses of the randomized controlled trials and only data regarding double-blind periods. For all outcomes, we extracted data for the intention-to-treat population.

### Risk-of-bias assessment

We performed a quality assessment using the Cochrane Collaboration’s tool for assessing the risk of bias in randomized trials, in which studies were scored as having a high, low, or unclear risk of bias in 5 domains: selection, performance, detection, attrition, and reporting biases [10]. Two independent authors conducted the bias evaluation without the use of automation tools, and disagreements were resolved by a third author. We did not evaluate small-study effect bias with a funnel plot due to the small number of included trials.

### Certainty assessment

Grading of Recommendations Assessment, Development, and Evaluation (GRADE) was employed to assess the level of certainty of the results (Additional file 1: Figure S1) [11]. For our analysis, we used GRADEpro software [12]. 

### Data synthesis and effect measures

This systematic review and meta-analysis was performed following the guidelines of the Cochrane Collaboration and the Preferred Reporting Items for Systematic Reviews and Meta-Analysis (PRISMA) guidelines [13]. Mean differences (MDs) with 95% confidence intervals (CIs) were used to compare treatment effects for continuous variables. Odds ratios (ORs) with 95% confidence intervals (CIs) were used to compare treatment effects for categorical endpoints. The Cochran Q test [100 × (*Q* – *df* ÷ *Q*)] and *I²* statistics were used to assess heterogeneity; *I² * > 25% was considered to indicate heterogeneity. We used a fixed-effects model for outcomes with low heterogeneity (*I²* < 25%). Otherwise, a DerSimonian and Laird random-effects model was used. The statistical analysis of the efficacy outcomes was carried out using R 4.1.0 (R Core Team, 2023), the *meta* and *metapower* packages. Review Manager 5.4 (Cochrane Centre, The Cochrane Collaboration, Denmark) was used for the statistical analysis of side effects. For the summary treatment effect estimate, a p value less than 0.05 was considered statistically significant.

## Results

### Database characteristics

**Fig. 1:**
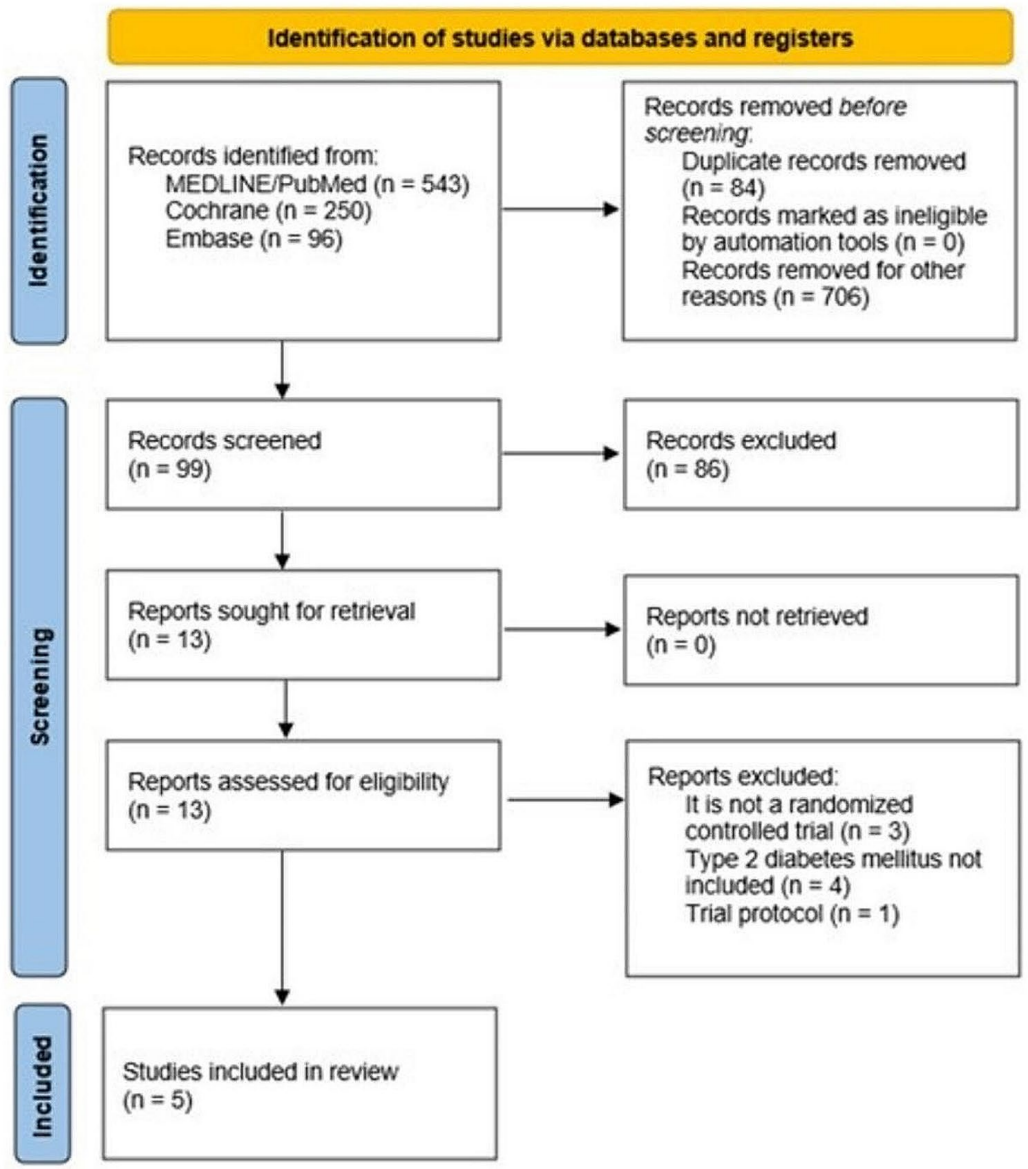
PRISMA flow chart for the identification, inclusion, and exclusion of studies

Our initial search resulted in 1119 entries. After deduplication and removal of studies that did not meet the inclusion criteria, the remaining 13 articles were fully reviewed. Finally, 5 randomized clinical trials were considered eligible, totaling 415 patients (Fig. 1). Additionally, data from a post hoc analysis of one of the studies included were considered. The follow-up period ranged from 5 to 26 weeks.

Two trials included liraglutide, one included exenatide, another included dulaglutide, and the last included lixisenatide. The trial sample size ranged from 21 to 154 patients. The follow-up period ranged from 5 to 26 weeks. The mean age of the population analyzed ranged from 14.5 to 15.8 years, with 66% being female. The baseline mean HbA1c averaged from 7.78 to 8.3%, the mean body weight ranged from 89 kg to 101 kg, and the mean body mass index ranged from 33.90 to 37.14. The mean duration of diabetes ranged from 1.6 to 3.5 years. Details of the studies and participants’ characteristics are presented in Table 1.

**Table 1:** Summary of the baseline characteristics of the participants included in the randomized controlled trials

	GLP-1 RA	Design	Sample size	Duration (weeks)	Female (%)	Mean age (years)	Mean HbA1c (%)	Mean body mass index (kg/m^2^)	Mean body weight (kg)
Arslanian 2022	Dulaglutide	Randomized, parallel-group, placebo-controlled, double-blind, multicenter, phase 3 superiority clinical trial.	154	26	71	14.5 ± 2.0	8.1 ± 1.3	34.1 ± 8.8	90.5 ± 26.5
Tamborlane 2022	Exenatide	Randomized, parallel-group, placebo-controlled, double-blind, multicenter, phase 3 clinical trial.	83	24	58.5	15 ± 1.8	8.2 ± 1.3	36.36 ± 8.57	100.6 ± 28.1
Tamborlane 2019	Liraglutide	Randomized, parallel-group, placebo-controlled, double-blind, multicenter, phase 3 clinical trial.	134	26	61.9	14.6 ± 1.7	7.78 ± 1.34	33.90 ± 9.25	91.5 ± 26.8
Barrientos-Pérez 2022	Lixisenatide	Randomized, parallel-group, placebo-controlled, double-blind, multicenter, phase 1 clinical trial.	23	6	69.57	15.56	8.16	34.11	92.76
Klein 2014	Liraglutide	Randomized, parallel-group, placebo-controlled, double-blind, multicenter clinical trial.	21	5	66.67	14.8 ± 2.2	8.1 ± 1.2	40	113.2 ± 35.6

**Fig. 2:**
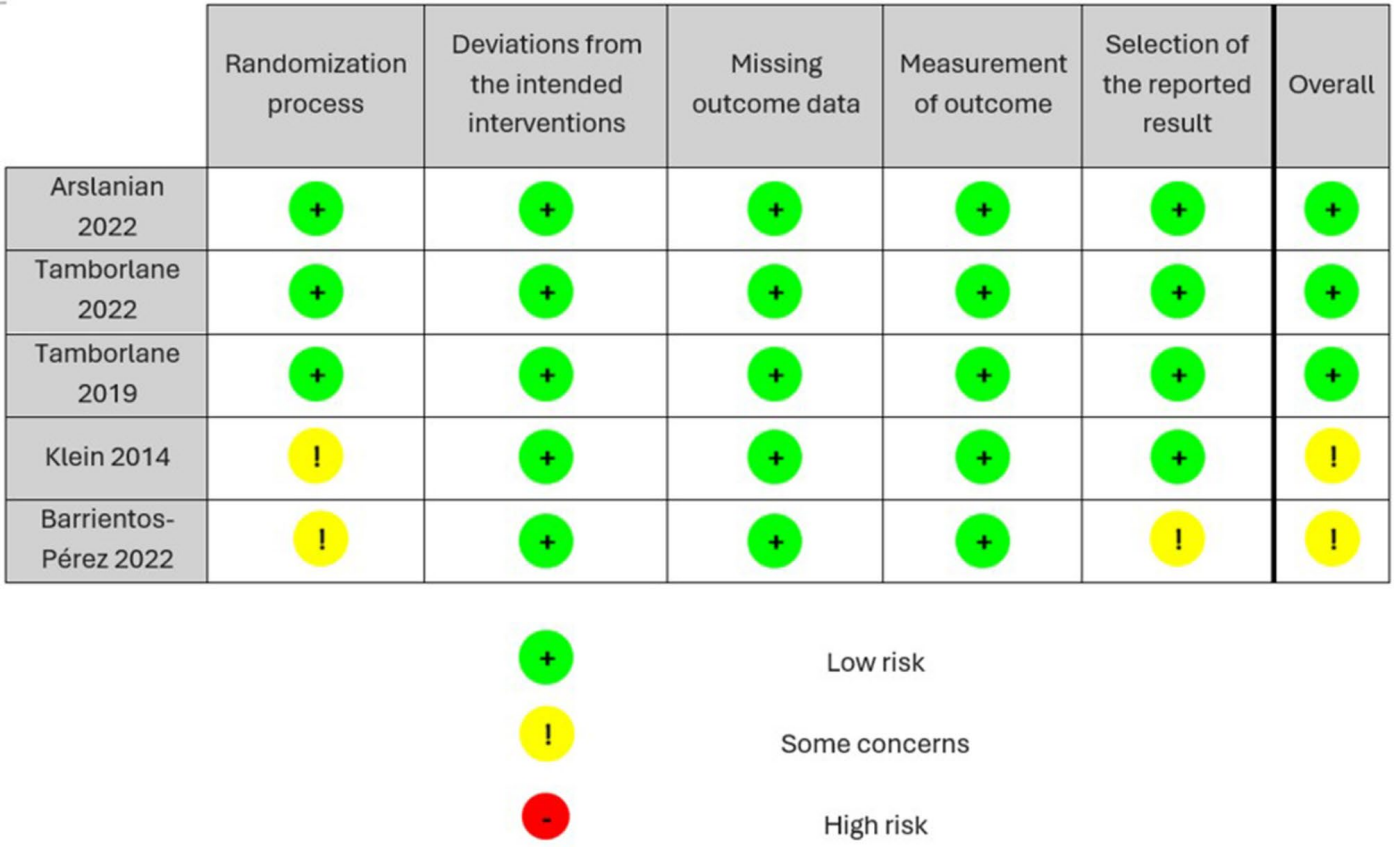
Risk of bias assessment of the included randomized controlled trials

After using the Cochrane Collaboration tool for assessing the risk of bias in the included studies, three of them were classified as low risk, and two showed some concerns about randomization due to small sample sizes. A risk of bias graph and a risk of bias summary were generated for the final analysis (Fig. 2). There were no studies with a high risk of bias.

Regarding the efficacy outcomes, the HbA1c and FBG data demonstrated a high level of evidence certainty, while body weight showed a moderate level. Both SBP and DBP showed a low certainty. Among the side-effect outcomes, hypoglycemia presented a high level of evidence certainty, as did most of the GI adverse effects. Abdominal pain data were the only data in this group that presented moderate certainty of evidence.

### HbA1C

**Fig. 3:**
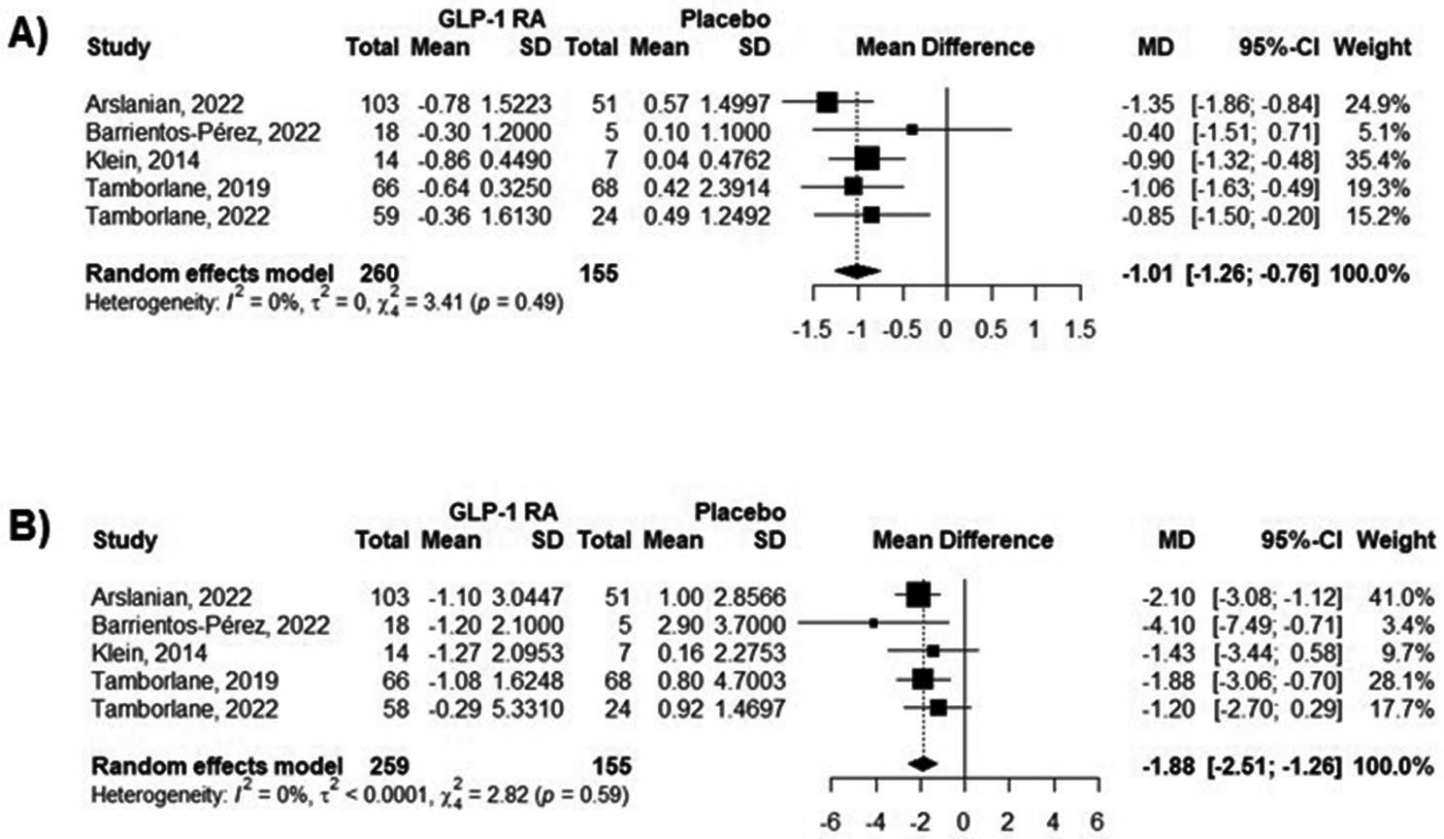
Forest plot from meta-analysis models of GLP-1 RA’s impact on (a) HbA1c and (b) fasting blood glucose. The data are presented as % and mmol/L, respectively

All studies we gathered presented data regarding the impacts of GLP-1 RA on HbA1c, showing reductions varying from − 0.3% to -0.86%. When comparing the intervention group with placebo through meta-analysis, GLP-1 RA lowered the HbA1c level by -1.01% (95% CI -1.26; -0.76), as presented in Fig. 3a.

### Fasting blood glucose

All 5 studies we collected reported the effects of GLP-1 RA on FBG, with reductions ranging from − 0.29 to -1.27 mmol/L. According to our meta-analysis comparing GLP-1 RA with placebo, the intervention lowered the FBG by -1.88 mmol/L (95% CI -2.51; -1.26) (Fig. 3b).

### Body weight

**Fig. 4:**
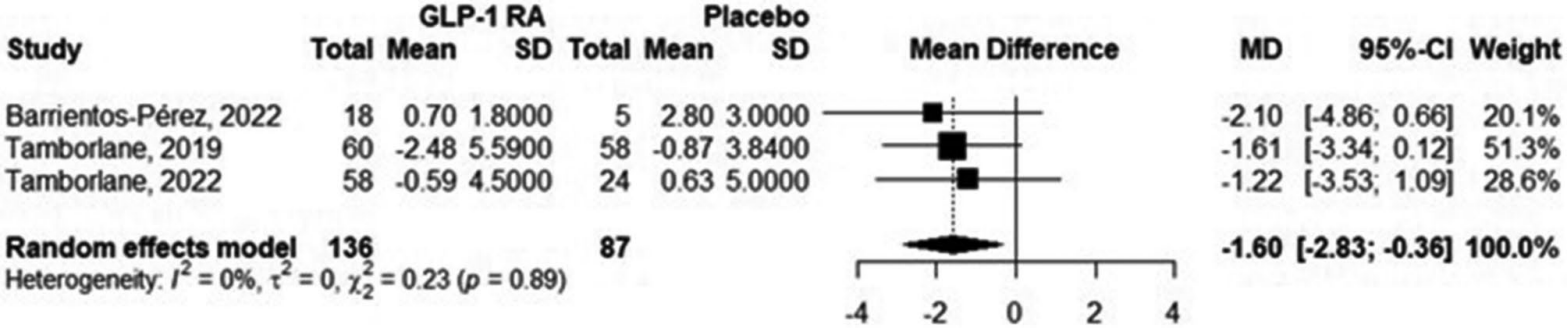
Forest plot from meta-analysis models of the impact of GLP-1 RA on body weight. The data are presented as kg

Three out of five studies reported data about body weight. The effects of therapy on this metabolic variable ranged from − 2.48 to + 0.7 kg. Meta-analysis revealed that compared with placebo, GLP-1 RA lowered weight by -1.6 kg (95% CI -2.83; -0.36), as shown in Fig. 4.

### Blood pressure

**Fig. 5:**
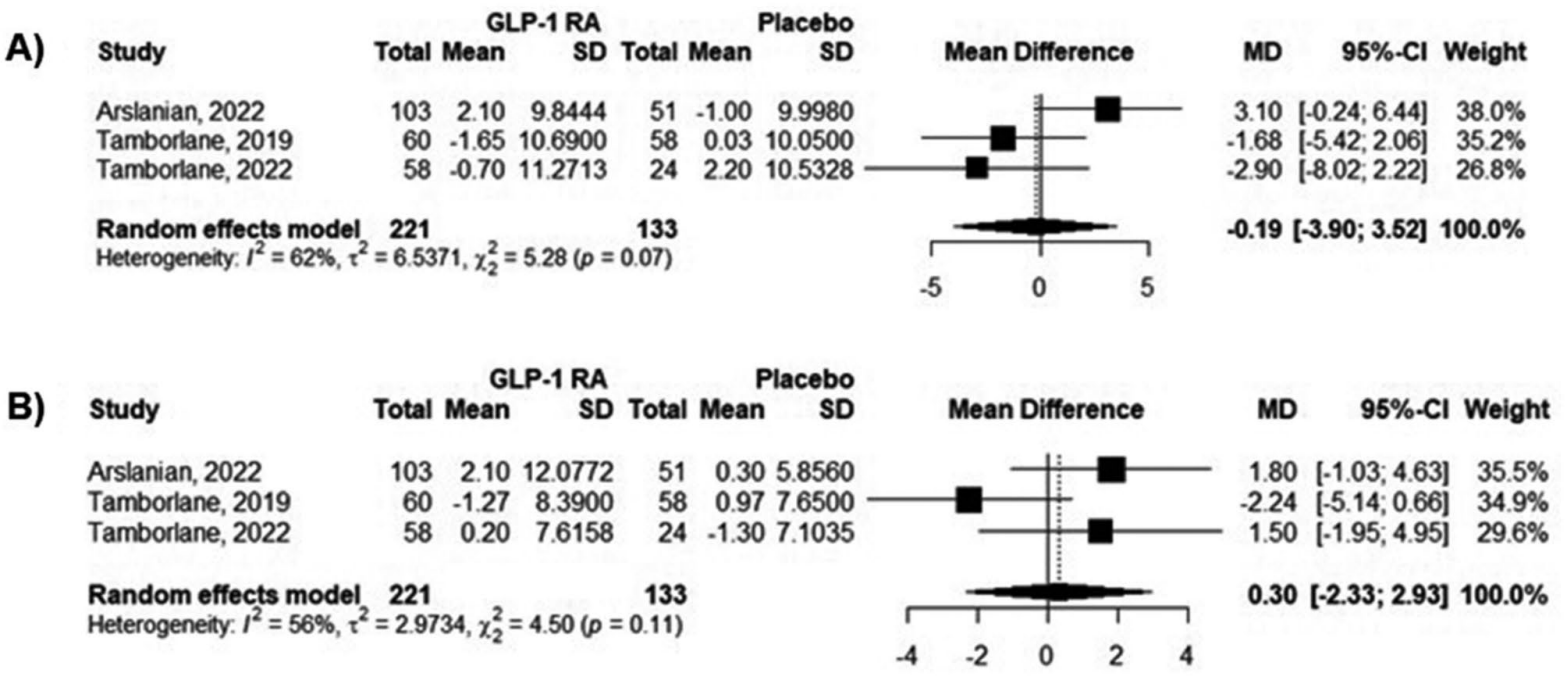
Forest plot from meta-analysis models of the impact of GLP-1 RA on (a) systolic and (b) diastolic blood pressure. The data are presented as mmHg

Only three of the included studies contained data regarding the blood pressure effects of GLP-1 RA. SBP (MD -0.19 mmHg [95% CI -3.9; 3.52 mmHg]) and DBP (MD 0.3 mmHg [95% CI -2.33; 2.93 mmHg]) were not statistically different between the intervention and placebo groups (Fig. 5a, b), respectively. Both SBP and DBP presented high statistical heterogeneity (I²) in our analysis (62% and 56%, respectively).

### Hypoglycemia

**Fig. 6:**
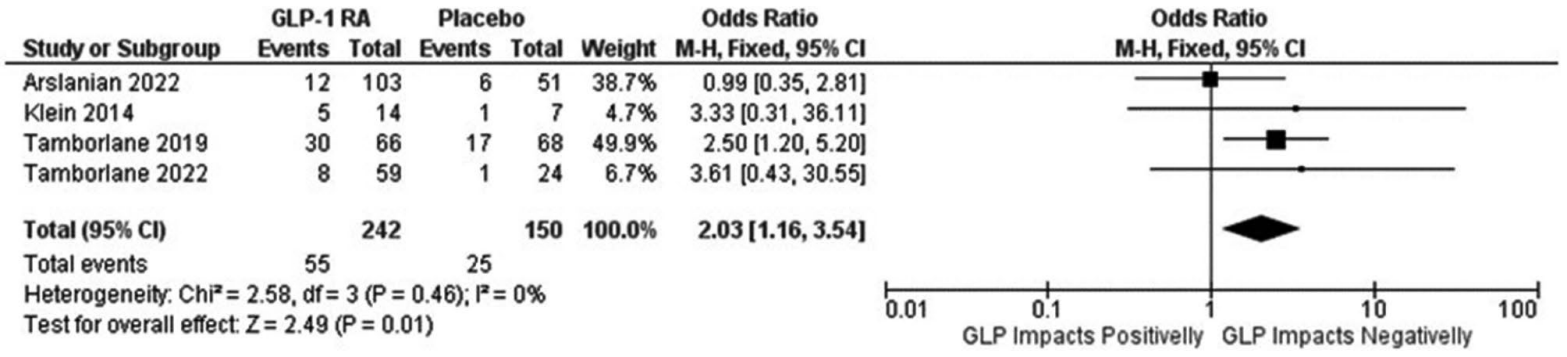
Forest plot from meta-analysis models of the impact of GLP-1 RA on any hypoglycemia

The incidence of any hypoglycemic episodes was defined as a plasma glucose concentration ≤ 3.9 mmol/L. Compared to the placebo group, the GLP-1 RA group demonstrated a greater likelihood of developing this condition (OR 2.03 [95% CI 1.16; 3.54]) (Fig. 6). One study was not included in the analysis because no data were available, and we did not conduct a meta-analysis specifically for severe hypoglycemia since events were rare.

Only one study excluded patients receiving insulin treatment at baseline [14]. Among the patients included, 106 were treated with insulin (with or without metformin). Fifty-seven patients in the intervention group received concomitant insulin treatment. One study included one patient taking metformin with sulfonylurea in the intervention group [6]. 

### Gastrointestinal adverse effects

**Fig. 7:**
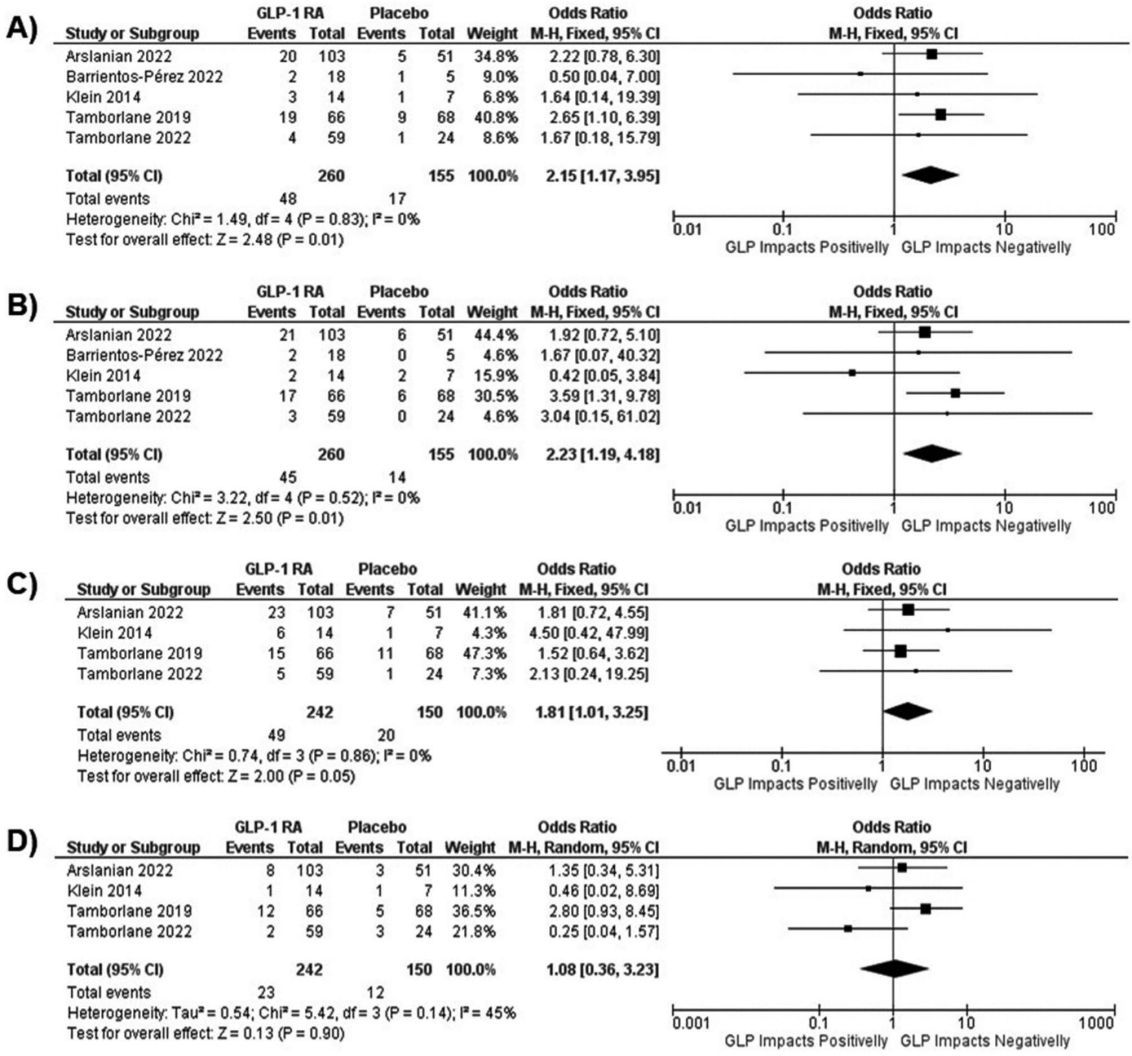
Forest plot from meta-analysis models of the impact of GLP-1 RA on (a) nausea, (b) vomiting, (c) diarrhea, and (d) abdominal pain

All five studies reported data regarding gastrointestinal adverse effects such as nausea and vomiting, and four of those reported diarrhea and abdominal pain. When comparing GLP-1 RA vs. placebo, patients in the intervention group had greater odds of developing adverse events. Nausea had an OR of 2.15 (95% CI 1.17; 3.95), and vomiting had an OR of 2.23 (95% CI 1.19; 4.18). Although inferior to the two previous adverse event outcomes, diarrhea presented an OR of 1.81 (95% CI 1.01; 3.25). The incidence of abdominal pain did not differ between the GLP-1 RA group and the placebo group (OR 1.08, 95% CI 0.36; 3.23). The forest plots of these results are presented in Fig. 7.

## Discussion

To the best of our knowledge, this is the first meta-analysis focused on the efficacy and safety of GLP-1 RA in pediatric patients with T2DM. Unlike previous meta-analyses that encompassed pediatric patients with obesity, which potentially compromised sample representativeness, our analysis reveals distinctive effect sizes. Although these studies align with adult findings, our examination exposes variations in effect sizes. It is uncertain whether one medication is superior to the other due to the insufficient number of trials with each GLP-1 RA to perform a subgroup or network meta-analysis. Moreover, some studies have a small population, which interferes drastically with confidence intervals in the original studies and with individual analysis. Our findings demonstrate that, on average, GLP-1 RA reduces HbA1c by 1%, fasting blood glucose by 1.88 mmol/L, and body weight by 1.6 kg. Notably, no significant reduction in SBP or DBP was observed. Despite a higher incidence of side effects, including hypoglycemia, vomiting, and diarrhea, associated with GLP-1 RA therapy, withdrawal rates from the studies remained low. Furthermore, the consistently low I² in most analyses indicates data accuracy.

The impact on HbA1c and body weight mirrors that observed in adults; however, these medications exhibit heightened efficacy in lowering fasting blood glucose in children [15, 16]. As anticipated, there is a lack of significance in DBP changes in children, paralleling the pattern observed in adults [17]. Conversely, SBP reductions are evident in adults but not in children, likely due to the low prevalence of hypertension in the latter [18, 19].

According to the findings from obesity meta-analyses, children with T2DM tend to lose weight to a lesser extent [20, 21]. Chadda et al. demonstrated a weight loss of 0.97 kg in children with T2DM, which was not statistically significant. In contrast, patients with obesity but without diabetes significantly lost 2.74 kg [20]. This parallels what is observed in adults with T2DM, potentially due to concurrent insulin use in some trials. Additionally, we observed a high risk for hypoglycemia, which mimics findings in adults, and we hypothesize that this may also be due to insulin use [22].

Lowering glycated hemoglobin stands as a paramount objective in the management of youth-onset T2DM. These young individuals confront an elevated risk of developing target organ damage, including diabetic retinopathy and kidney disease, along with a heightened mortality rate compared to their counterparts with type 1 diabetes [4, 23, 24]. However, attaining glycemic control poses a formidable challenge in children and adolescents with T2DM [25]. The onset of the disease during youth is correlated with higher rates of metformin monotherapy failure and a less favorable glycemic trajectory compared to adult-onset disease [26].

Presently, American guidelines advocate for the use of GLP-1 receptor agonists as second-line treatments for both children and adults for primary prevention of macrovascular disease [9, 27–29]. The paucity of evidence has not only curtailed the indication of GLP-1 RA but has also confined it to specific pharmaceuticals within this age group. In the United States, approval has been granted for liraglutide and extended-release exenatide for pediatric patients aged ≥ 10 years with T2DM [30, 31]. Conversely, within the United Kingdom, sole approval is accorded to liraglutide; however, clinicians frequently opt for dulaglutide due to constraints on available alternatives [32]. In this context, our meta-analysis supports the efficacy and safety of GLP-1 receptor agonists, proffering viable therapeutic options for the amelioration and prevention of complications associated with pediatric T2DM.

Our meta-analysis exhibits certain limitations. The dearth of randomized controlled trials involving children, most of which have small sample sizes, may be attributed to the lower prevalence of T2DM in children than in adults, thereby compromising patient recruitment. There are more active randomized clinical trials that could contribute to the current knowledge of our topic; however, they have not published results yet. (NCT04596631, NCT00658021, NCT04873245). Additionally, throughout the data retrieval process, we encountered several challenges pertaining to the manner in which numerical data were reported in the articles encompassed within our study. This issue is particularly disconcerting, as the provision of clear, comprehensible, and reproducible data is imperative for informed clinical decision-making. This challenge is further exacerbated when dealing with small populations, necessitating the maximal utilization of available data. Furthermore, our study predominantly comprises females and Caucasians, whereas the disease predominantly affects indigenous and black populations [2, 33, 34]. To enhance the generalizability of the results, future studies should encompass more diverse populations with clear and accessible data. Such inclusivity is imperative for better informing clinical practice, especially when tending to vulnerable patients with a significant risk of comorbidities. Physicians rely heavily on this information to guide their clinical decisions.

In conclusion, this meta-analysis provides precise data pertaining to the efficacy and safety profile in children with T2DM. Within this specific population, GLP-1 RA exhibits a notable association with substantial reductions in HbA1c, FBG, and body weight. The administration of these medications is concurrent with an elevated incidence of side effects, which are predominantly gastrointestinal and deemed tolerable.

### Electronic supplementary material

Below is the link to the electronic supplementary material.

Supplementary Material 1

## Data Availability

No datasets were generated or analysed during the current study.

## Abbreviations

GLP-1: glucagon-like peptide 1
GLP-1 RA: glucagon-like peptide 1 receptor agonist
T2DM: type 2 diabetes mellitus
MD: mean difference
OR: odds ratio
CI: confidence interval
HbA1c: glycated hemoglobin
FBG: fasting blood glucose
SBP: systolic blood pressure
DBP: diastolic blood pressure
GRADE: Grading of Recommendations Assessment, Development, and Evaluation
PRISMA: Preferred Reporting Items for Systematic Reviews and Meta-Analysis
