# Supplementary material for: The efficacy and safety of GLP-1 receptor agonists in youth with type 2 diabetes: a meta-analysis
Source: Diabetol Metab Syndr. 2024 Apr 24;16:92. doi: 10.1186/s13098-024-01337-5 (PMC11044464; doi:10.1186/s13098-024-01337-5)
Supplement: Supplementary file 1 — Supplementary Material 1 [file 13098_2024_1337_MOESM1_ESM.docx]

**Additional file 1**

**Table S1.** Search terms and number of records by each database

**Figure S1.** GRADE assessment of the outcomes of the meta-analysis

**Table S1. Search terms and number of records by each database**

| **Search terms** | ‘diabetes mellitus type 2’, ‘child’, ‘children’, ‘adolescent’, ‘youth’, ‘teen’, ‘teenager’, ‘juvenile’, ‘dulaglutide’, ‘semaglutide’, ‘liraglutide’, ‘exenatide, ‘lixisenatide’, ‘GLP-1 analog’, ‘glucagon-like peptide 1 receptor agonist’, and ‘randomized controlled trial’ |
| --- | --- |
| **Database** | **Number of records** |
| MEDLINE/PubMed | 676 |
| Embase | 346 |
| Cochrane | 97 |

Search terms and summary of records found by database.

**Figure S1. GRADE assessment of the outcomes of the meta-analysis.**


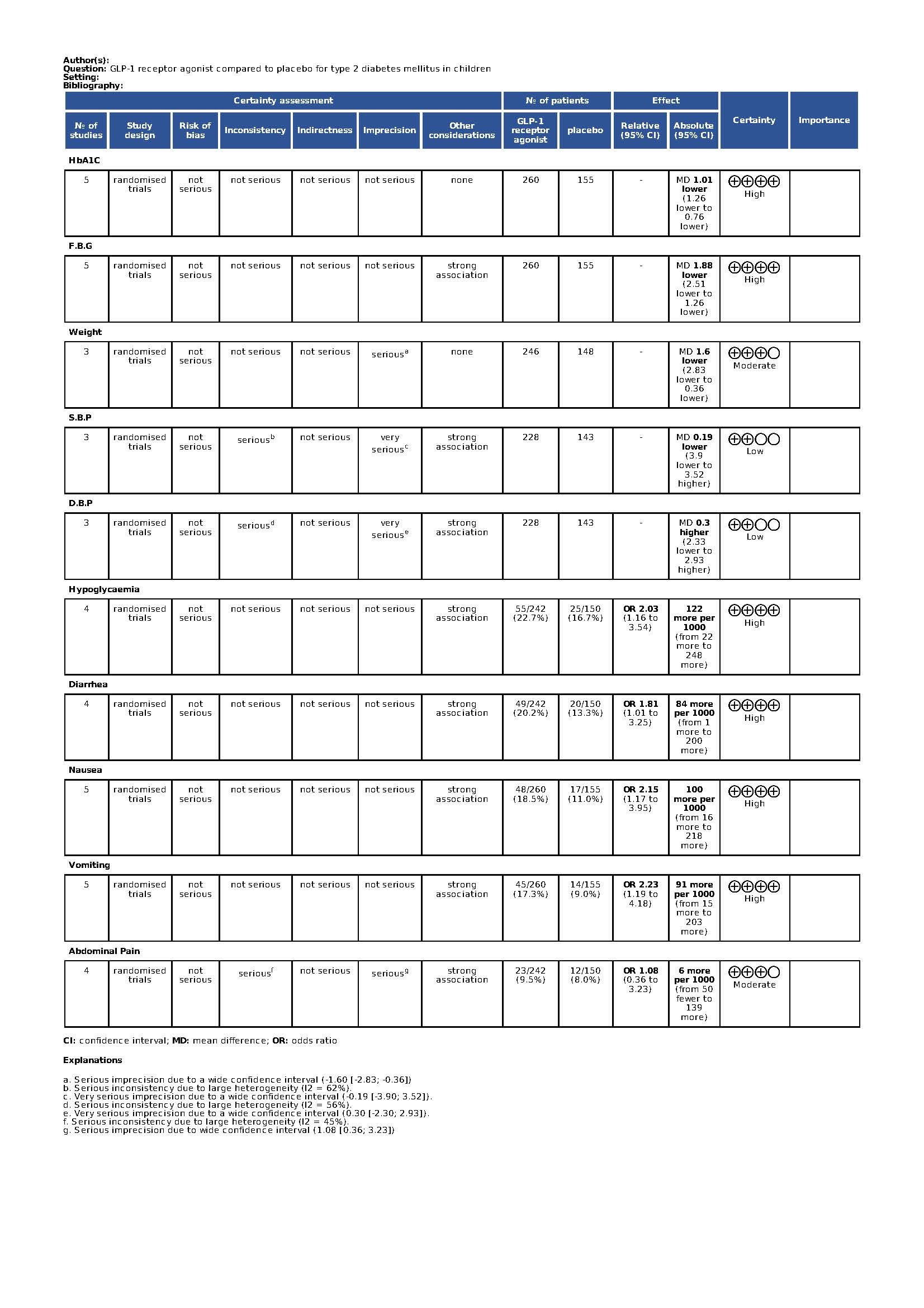


Summary of certainty of evidence assessment for all outcomes.
